# The Intestinal Microbiota in Metabolic Disease

**DOI:** 10.3390/nu8040202

**Published:** 2016-04-06

**Authors:** Anni Woting, Michael Blaut

**Affiliations:** Department of Gastrointestinal Microbiology, German Institute of Human Nutrition Potsdam-Rehbruecke, Arthur-Scheunert-Allee 114-116, 14558 Nuthetal, Germany; blaut@dife.de

**Keywords:** intestinal microbiota, obesity, diabetes, metabolic syndrome, energy harvest, diet, absorption, bile acids, low-grade inflammation, SCFA

## Abstract

Gut bacteria exert beneficial and harmful effects in metabolic diseases as deduced from the comparison of germfree and conventional mice and from fecal transplantation studies. Compositional microbial changes in diseased subjects have been linked to adiposity, type 2 diabetes and dyslipidemia. Promotion of an increased expression of intestinal nutrient transporters or a modified lipid and bile acid metabolism by the intestinal microbiota could result in an increased nutrient absorption by the host. The degradation of dietary fiber and the subsequent fermentation of monosaccharides to short-chain fatty acids (SCFA) is one of the most controversially discussed mechanisms of how gut bacteria impact host physiology. Fibers reduce the energy density of the diet, and the resulting SCFA promote intestinal gluconeogenesis, incretin formation and subsequently satiety. However, SCFA also deliver energy to the host and support liponeogenesis. Thus far, there is little knowledge on bacterial species that promote or prevent metabolic disease. *Clostridium ramosum* and *Enterococcus cloacae* were demonstrated to promote obesity in gnotobiotic mouse models, whereas bifidobacteria and *Akkermansia muciniphila* were associated with favorable phenotypes in conventional mice, especially when oligofructose was fed. How diet modulates the gut microbiota towards a beneficial or harmful composition needs further research. Gnotobiotic animals are a valuable tool to elucidate mechanisms underlying diet–host–microbe interactions.

## 1. Introduction

Recent years have seen a surge in publications reporting correlations between the gut microbiota and various medical conditions such as inflammatory bowel disease, colorectal cancer, allergies, autism and kidney stones. This development has been fostered by considerable technological progress and the advent of the omics technologies, which afford a fast and relatively inexpensive culture-independent assessment of complex microbial communities, their gene repertoire (the microbiome), gene expression and metabolic profiles. The role of intestinal bacteria in the development of obesity and associated diseases has attracted particular attention, not only in the scientific community but also in the lay press, because it has become a major public health issue. Even though obesity and metabolic disease are considered to be nutrition-related disorders, recent evidence indicates that the intestinal microbiota plays a major role in disease development.

## 2. Intestinal Microbiota

The gut microbiota of a given animal species has co-evolved with its host such that it is optimally adapted to the intestinal environment of the respective host. Thus, it is not surprising that the microbial community inhabiting the digestive tract affects host physiology in many ways, mainly by interacting with the host immune system and by broadening the metabolic potential of the host. The majority of microorganisms in the gut are considered commensals, as they perform tasks that are beneficial for the host. However, even though this microbial community usually lives in harmony with its host, it should be kept in mind that gut bacteria are not altruistic but solely taking advantage of the constant temperature and the wide range of substrates available in the digestive tract. In return, by virtue of its immense metabolic potential, the intestinal microbiota makes otherwise non-utilizable nutrients available to the host. For example, non-digestible carbohydrates, also referred to as dietary fiber, are fermented to short-chain fatty acids (SCFA), which can be utilized by the host. However, under certain circumstances, the harmonic relationship between the host and its microbiota gets lost. Possible reasons include medication, a diseased state and/or unhealthy nutrition. Interestingly, various diseases are accompanied by alterations in the gut microbiota, often referred to as dysbiosis.

### 2.1. Composition

The gut microbiota of adult healthy subjects is dominated by six bacterial phyla: Firmicutes, Bacteroidetes, Proteobacteria, Actinobacteria, Fusobacteria and Verrucomicrobia. Besides these phyla of the domain Bacteria, the human digestive tract also harbors the methanogen *Methanobrevibacter smithii* (*M. smithii*), which belongs to the Archaea domain and can be found in every second individual, as well as eukaryotic organisms, such as the yeast *Candida*. While methanogens and yeasts contribute to less than 1% of all microbial cells of the fecal microbiota, Firmicutes and Bacteroidetes together can reach a proportion of more than 90%, while the proportion of representatives of the other phyla ranges from 2% to 10% [1]. A comparison of data from various mouse studies indicates that the proportions reported for the different phyla differ quite considerably among these studies. For example, while the proportion of Actinobacteria in the study by Hildebrandt *et al.* was less than 1% [2], Murphy *et al.* reported Actinobacteria to make up 11%–25% [3]. These inter-study discrepancies are probably to a large extent due to differences in the protocols used for sampling, storage and DNA extraction. Indeed, a recent study demonstrated that the DNA extraction method is critical for the detection of clostridial and actinobacterial populations [4]. Therefore, protocols have to be rigorously tested and harmonized to make studies more comparable.

Most knowledge about the composition of the human gut microbiota stems from the analysis of fecal samples. However, it has to be kept in mind that microbiological data based on such analyses are not representative of the situation in the various gut sections. Moreover, there are considerable differences between the microbial communities in the gut lumen and those adhering to the mucus layer covering the intestinal mucosa [5].

### 2.2. Key Activities of the Gut Microbiota

Substrate availability and physicochemical conditions in the intestinal tract are key factors that affect the composition and activity of the gut microbiota. The majority of substrates required by intestinal bacteria for their growth stems from the diet. In addition, the host provides mucins, desquamated epithelial cells and digestive enzymes as additional substrates to intestinal bacteria. Important dietary substrates for gut bacteria include undigested or incompletely digested carbohydrates such as resistant starch and dietary fiber. The latter includes a large variety of carbohydrate polymers typically found in dietary plants. Cellulose, hemicellulose and pectin are components of the plant cell wall, whereas inulin is used by some plants for carbohydrate storage. In contrast to humans, who are devoid of enzymes capable of breaking down these carbohydrate polymers, the intestinal microbiome encodes a broad spectrum of enzymes that catalyze their depolymerization and further degradation. In fact, carbohydrate degradation pathways are over-represented in the human gut microbiome compared to other microbial genomes [6]. The underlying bacterial activities enable the host to take advantage of indigestible carbohydrates that otherwise would be excreted unused. The enzymes provided by the gut microbiome afford the depolymerization of a wide range of carbohydrates such as xylans, α- and β-glucans, fructans, β-mannans and pectins [7]. Intestinal bacteria involved in this process include members of both the Firmicutes (*Ruminococcus, Butyrivibrio* and *Roseburia* species) and the Bacteroidetes (*Bacteroides* spp.). The starch utilization system (Sus) from *Bacteroides thetaiotaomicron* (*B. thetaiotaomicron*) has been studied in detail [8,9] and been used as the paradigm for other polysaccharide degradation systems in *Bacteroides* spp. Much less is known about the carbohydrate utilization systems in members of the Firmicutes, even though these bacterial species play a major role in colonic carbohydrate fermentation [7,10].

Mucus produced by goblet cells represents another important source of growth substrates for intestinal bacteria. It can be utilized by a considerable number of intestinal bacteria, including *B. thetaiotaomicron* [11], *Bifidobacterium bifidum* [12], and *Akkermansia muciniphila* [13], a member of the phylum Verrucomicrobia*.* Intestinal mucins consist of up to 80% of glycans attached to a protein backbone, which accounts for approximately 20% of the molecule. *B. thetaiotaomicron* is one of the most versatile glycan utilizers in the intestine. However, this bacterium does not utilize various glycans simultaneously, but rather prioritizes their utilization, regardless of their dietary or host-derived origin [14]. This is accomplished by a highly sophisticated regulatory network, which involves hybrid two-component systems. The latter are transmembrane proteins consisting of two domains, which in classical bacterial two-component systems are made up of two separate proteins. The periplasmic domain of this transmembrane protein acts as a glycan sensor while the cytoplasmic domain contains a helix-turn-helix DNA-binding module that controls the expression of genes involved in glycan utilization [15,16].

Even though the majority of intestinal micro-organisms prefer glycans over proteins as growth substrates, proteins are also utilized, in particular in the distal colon where the availability of carbohydrates is limited because they have been used up in the proximal part of the intestinal tract [17]. The amount of dietary protein that reaches the colon is small but not negligible. In addition, digestive enzymes and desquamated epithelial cells are another protein source for colonic bacteria. Proteins reaching the colon are first cleaved into peptides and amino acids, which undergo further bacterial degradation. Bacterial amino acid degradation in the colon involves oxidative and reductive deamination reactions often followed by decarboxylations resulting primarily in the formation of SCFA. Further typical degradation products include amines, branched-chain fatty acids produced from iso-amino acids as well as phenolic and indolic compounds produced from aromatic amino acids [18,19,20].

Other activities of the intestinal microbiota include the conversion of secondary plant metabolites such as glucosinolates in Brassica vegetables [21,22] or polyphenols in fruits, vegetables, cereals, chocolate, tea, coffee, or wine [23]. Some transformation products formed by intestinal bacteria may have health-promoting properties and have therefore been a major topic in nutrition research.

Intestinal bacteria also play a role in the metabolism of xenobiotics and the conversion of bile acids. Xenobiotics are first oxidised and subsequently sulphated or glucuronidated to render them water soluble and thereby facilitate their urinary excretion. Following their synthesis in the liver, bile acids (cholic acid and chenodeoxycholic acid in humans) are conjugated with either glycine or taurine and then secreted into the intestinal tract, where they undergo deconjugation and partial dehydroxylation by intestinal bacteria [24].

In contrast, lipids cannot be utilized by anaerobic bacteria because the oxidation of long-chain fatty acids requires the presence of oxygen, which is scarce in the intestine. Therefore, changes in the gut microbiome observed in response to high-fat diets are rather due to changes in the intestinal environment. For example, a rat study revealed that oral administration of the bile acid cholate induced changes in the composition of the gut microbiota similar to those observed after feeding a high-fat diet [25], indicating that diet-related microbiota changes may be of indirect nature (see section 4 for more details).

## 3. Obesity and Metabolic Disease and Their Link to the Intestinal Microbiota

Obesity is often accompanied by dyslipidemia, hypertension and impaired glucose homeostasis, known as metabolic syndrome. The consumption of energy-dense foods as well as the low energy requirement for physical activity and reproduction are the main determinants of obesity in Western countries. In the last decade the intestinal microbiota has been proposed as another environmental factor involved in the onset of obesity. However, to which extent and through which mechanism the intestinal microbiota contributes to obesity development has not yet been elucidated.

A comparison of germfree and conventional mice revealed that the intestinal microbiota contributes to an obese phenotype [26,27,28,29]. However, the conclusion that germfree C57BL/6 mice are generally protected from diet-induced obesity as reported by Backhed *et al.* (2007) could not be reproduced for all mouse strains and diets. Interestingly, germfree C3H mice were protected from obesity when fed the same Western diet used by Backhed and colleagues, whereas the feeding of a semi-synthetic high-fat diet with essentially the same macronutrient composition but other ingredients increased the body weight gain in these mice [30]. These and other discrepancies in the literature call for a better understanding of the interplay between diet and host health and for an elucidation of the exact role of gut bacteria in this interaction.

That the intestinal microbiota plays an important role in obesity development can be deduced from fecal transplantation experiments. Transplantation of fecal microbiota from obese mice to lean germfree mice also transferred the obese phenotype to the recipients. Mice that received the gut microbiota from lean mice stayed lean [31,32]. The adipose phenotype was even transmissible from human twins discordant for obesity to germfree mice by a single oral gavage of feces [33]. However, co-housing of mice that received the lean or the obese microbiota prevented increased adiposity in the recipients of the obese microbiota. Members of the Bacteroidetes present in the lean microbiota successfully invaded the obese microbiota when the mice were fed a low-fat, fiber-rich diet. This was accompanied by a reduction of body fat in the mice that originally received the microbiota from the obese donors. However, the invasion of Bacteroidetes and the prevention of adiposity failed when the mice consumed a high-fat, low-fiber diet [33]. This study demonstrates the close relationship between diet and intestinal microbiota since the microbial composition is modifiable by diet with direct consequences for host health.

## 4. Impact of Energy-Rich Diets on Microbiota

**Figure 1:**
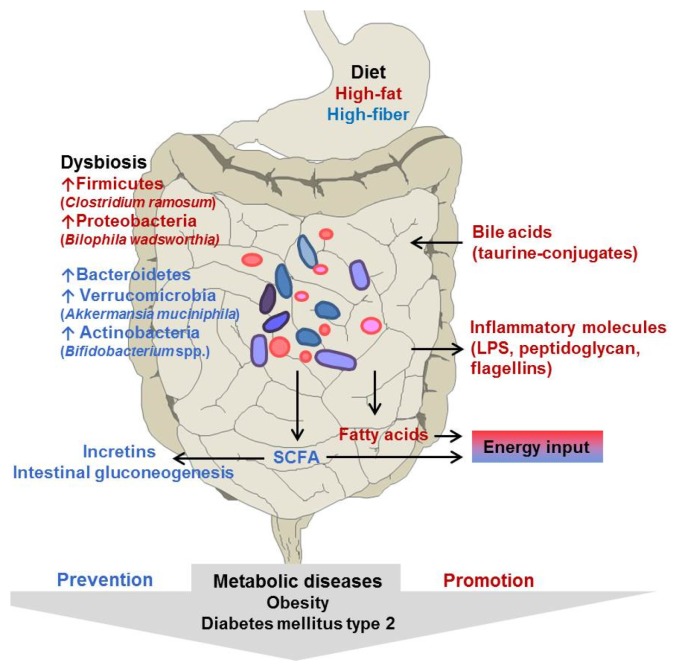
Hypothetical interplay between diet, gut microbiota and host in prevention and promotion of metabolic diseases. Consequences of high-fat diets and fiber-rich diets are indicated in red and in blue, respectively.

Dietary modifications change the intestinal microbiota of mice and humans within one day [32,34,35]. Such diet-induced changes in the gut microbiota are hypothesized to promote the development of obesity and associated chronic diseases. Diet selects for certain bacteria as shown by Ridaura *et al.* [33], but in which way dietary components exactly lead to the observed changes in the microbiota is largely unknown (Figure 1).

Dietary intervention studies in mice revealed an increase in Firmicutes and a decrease in Bacteroidetes in obese individuals [2,3,36]. These studies suggest that the observed microbiota changes in obese mice were caused by diet rather than by the obese phenotype. The high proportion of Firmicutes in obese mice fed high-fat diets was in part due to the proliferation of Erysipelotrichi, a bacterial class within this phylum, formerly known as Mollicutes [30,32,37]. However, genetically obese mice also harbor more Firmicutes and correspondingly less Bacteroidetes in their gut compared to their lean siblings [38], indicating that diet-independent host factors also modulate the microbiota.

In humans, obesity and the metabolic syndrome are also associated with a higher intestinal Firmicutes/Bacteroidetes ratio in comparison with lean or “healthy obese” individuals [39,40,41]. The consumption of calorie-restricted diets was accompanied by a reduction of body weight, and a shift from the high Firmicutes/Bacteroidetes ratio to a lower value typical of lean subjects [39]. Energy-rich diets were reported to increase the proportion of intestinal Firmicutes in both humans and mice, suggesting that dietary ingredients or endogenous metabolites secreted into the gut lumen in response to these diets (e.g., bile acids) were responsible for this phenomenon. However, other human trials not only failed to confirm a high proportion of Firmicutes in obese patients [42] but reported even the opposite [43]. The reasons for this discrepancy are not really known.

Amount and type of dietary fat affect the spectrum of bile acids formed in the liver and released into the intestine, which in turn influences the gut microbiota. For instance, a diet rich in saturated fatty acids promotes the hepatic production of taurine-conjugated bile acids at the expense of glycine-conjugated bile acids. The latter were dominant when an iso-caloric diet rich in polyunsaturated fat was fed [44]. Deconjugation of the taurine-conjugated bile acids stimulated the growth of *Bilophila wadsworthia* (*B. wadsworthia*) because this organism is able to gain sulfite from the taurine and to use it as an electron acceptor [44].

The administration of the primary bile acid cholic acid decreased the total bacterial cell count in the caecum of rats [25]. The authors proposed that microbial 7α-dehydroxylation of cholic acid to deoxycholic acid reduced the number of bacterial cells because deoxycholic acid has a 10 times higher bactericidal activity compared with cholic acid. Similar to the shifts at phylum level in response to high-fat feeding in mice [2,3,32,37], cholic acid-fed rats displayed an increase of caecal Firmicutes and a decrease of Bacteroidetes. The expansion of the Clostridia and Erysipelotrichi was mainly responsible for the high proportion of Firmicutes in these rats. The authors speculated that high dietary fat intake promoted the formation of deoxycholic acid, which in turn affected microbiota composition [25].

In humans who consumed an animal-based diet rich in dietary fat and protein, the gut microbiota was enriched with bile-tolerant taxa [35]. Similar to the changes in microbiota composition observed by Devkota *et al.* in mice in response to a diet rich in saturated fatty acids, the animal-based diet consumed by humans also promoted the expansion of *B. wadsworthia* in their intestinal microbiota [35]. It may be speculated that in both studies elevated intestinal bile acid concentrations promoted the outgrowth of this sulfite-reducing bacterium, which triggers colitis in genetically susceptible interleukin-10 knockout mice [44].

## 5. Influence of Intestinal Bacteria on Host Ability to Harvest Energy from the Diet

Bacterial degradation of non-digestible dietary polysaccharides to monosaccharides and their ensuing fermentation to SCFA by the intestinal microbiota is hypothesised to contribute to obesity development. A previous animal study indicated a link between high intestinal SCFA levels and obesity: Mice di-associated with *B. thetaiotaomicron* and *M. smithii* displayed higher caecal acetate concentrations, increased *de novo* lipogenesis and a higher epididymal white adipose tissue weight than germfree mice or mice mono-associated with either one of these strains [45]. Moreover, the metagenome of obese mice fed a Western diet was enriched in genes involved in the fermentation of simple sugars. Accordingly, the obese mice displayed elevated caecal SCFA concentrations [37]. In line with the notion that intestinal SCFA formation promotes overweight, a high-fat diet supplemented with a fermentable fiber fed to mice resulted in a higher body weight gain than the same high-fat diet except that the fermentable fiber had been replaced by a non-fermentable fiber [46]. This suggests that intestinal SCFA promoted body weight gain in the mice fed the fermentable fiber by delivering additional energy. In support of this explanation, obese mice fed high-fat diets poor in fermentable fiber displayed lower fecal acetate concentrations compared with lean mice fed a diet rich in fermentable fiber. However, the high acetate levels in the lean mice suggest that this lipogenic SCFA does not necessarily lead to obesity [30].

In humans, the consumption of diets rich in dietary fiber is associated with higher fecal SCFA concentrations as well as a lower incidence of obesity and symptoms of metabolic disease [47,48,49]. Therefore, dietary fiber is generally regarded as health conducive as it helps in the management of metabolic disease supposedly by virtue of the SCFA derived thereof by bacterial fermentation. However, human studies reported higher fecal SCFA levels in overweight and obese patients than in lean subjects, which was unlikely to be caused by a reduced colonic SCFA absorption or a higher intake of dietary fiber in the diseased subjects [43,50]. Similarly, in obese women who consumed an energy-dense diet, fecal SCFA concentrations correlated positively with adiposity and insulin resistance. However, the increased fecal SCFA levels could not be explained by the subjects’ fiber consumption [51]. The discrepancies observed between reduced fiber intake and elevated fecal SCFA concentrations are difficult to reconcile and might have as yet unknown reasons. For example, it is conceivable that the role of intestinal SCFA in obesity development depends on dietary factors other than the amount of consumed fiber.

It is also worth mentioning that dietary fiber does not always change the dominant microbial groups and the concentrations of SCFA in the gut [52]. Hence, fibers may exert beneficial effects on the host independently from the microbiota and from SCFA formation. This may in part be due to the fact that dietary fiber decreases the energy density of food and/or impairs absorption of amino acids and small peptides in the upper digestive tract [53]. Subsequently, the diminished amino acid supply could prevent the amino acid induced increase in expression of ribosomal protein S6 kinase beta-1 in subcutaneous adipose tissue that would otherwise have promoted insulin resistance. Nonetheless, the beneficial effects of dietary fiber may at least in part be mediated by SCFA as they have been demonstrated to modify host energy metabolism (see section 8 for more details).

## 6. Influence of Intestinal Bacteria on Monosaccharide Absorption

The human diet is rich in carbohydrates including complex polysaccharides, disaccharides and monosaccharides. Simple sugars such as glucose and fructose are rapidly absorbed, while disaccharides such as maltose, sucrose and lactose have to be hydrolyzed to monosaccharides prior to absorption in the small intestine. Polysaccharides such as hemicellulose, pectin and resistant starch escape digestion in the upper digestive tract. In the ileum, processing of these polysaccharides by bacterial glycosidases delivers monosaccharides, which may contribute to the energy demand of the host. A comparison of conventionalized mice and germfree mice revealed that the presence of a microbiota improves intestinal glucose absorption [26]. Conventionalized mice displayed increased levels of serum glucose and serum insulin, both of which are known activators of the transcription factors carbohydrate-responsive element-binding protein (ChREBP) and sterol regulatory element-binding protein 1 (SREBP1), respectively. Activation of the acetyl-CoA carboxylase gene (*Acac1*) and the fatty acid synthase gene (*Fasn*) by these transcription factors was proposed to promote hepatic *de novo* lipogenesis in the conventionalized mice. However, which bacterial species mediated the increased intestinal glucose absorption was not reported.

**Figure 2:**
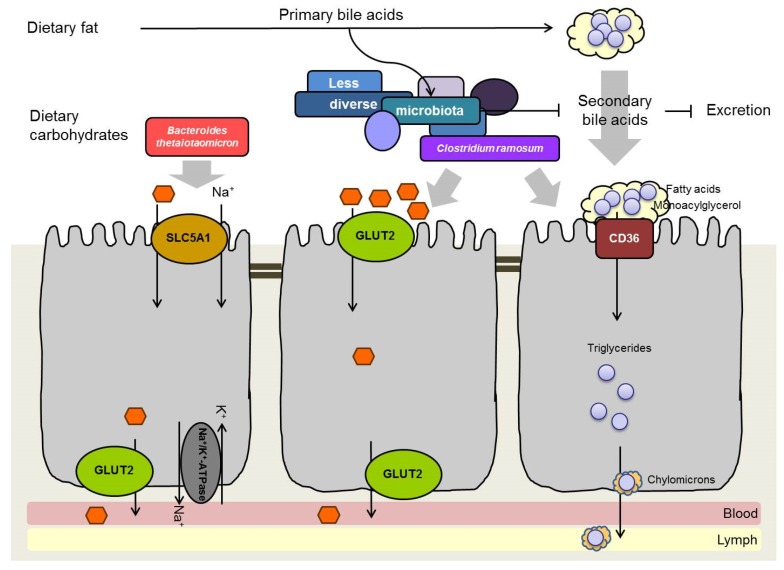
Hypothetical scheme displaying possible contributions of intestinal microbiota to obesity development.

A possible mechanism how gut bacteria facilitate glucose uptake might be an increased expression of glucose transporters. In support of this explanation association of germfree mice with *B. thetaiotaomicron* increased the ileal transcription of the sodium/glucose co-transporter 1 (*Slc5a1*). This transporter mediates glucose and galactose uptake in symport with sodium ions into enterocytes [54]. Interestingly, the presence of *Clostridium ramosum* (*C. ramosum*) in gnotobiotic mice increased the gene expression of the passive glucose transporter 2 (*Glut2*) in jejunum and ileum, but not that of *Slc5a1* [55] (Figure 2). However, further studies including the measurement of glucose fluxes are needed to assess the impact of microbes on monosaccharide absorption and the ensuing consequences for the development of metabolic diseases in the host.

## 7. Influence of Intestinal Bacteria on Lipid Absorption

Bile acids are synthesized in the liver and secreted into the small intestine where they solubilize dietary lipids through micelle formation. The emulsification increases the surface of the lipids and makes them accessible to lipolytic enzymes that cannot enter lipid droplets. Thereby, bile acids promote the cleavage of lipids resulting in the liberation of monoacylglycerol and fatty acids, which are subsequently absorbed by enterocytes. Bacterial deconjugation and dehydroxylation of bile acids lead to the formation of secondary bile acids, which are less effectively reabsorbed from the ileum and therefore excreted to a greater extent than primary bile acids [56,57]. These modifications catalyzed by the gut microbiota change the physicochemical properties of the bile acids. Possible consequences for the host include a less efficient micelle formation and a diminished lipid digestion and absorption [58]. Therefore, it may be hypothesized that the less diverse intestinal microbiota reported for obese subjects produces less secondary bile acids (deconjugated, dehydroxylated) and consequently, that high concentrations of primary bile acids promote dietary lipid emulsification, digestion and absorption (Figure 2). Indeed, microbial transformation of bile acids is weaker in obese than in lean mice [33]. However, whether bile acid transformation by bacteria in the small intestine causes diminished lipid absorption is doubtful.

Indeed, experiments by Rabot *et al.* (2010) are in conflict with this explanation because they showed that the intestinal microbiota promotes lipid absorption: High-fat diet-fed conventional mice excreted 40% less lipids in their feces than high-fat diet-fed germfree mice [29], the opposite of what would have been expected if conjugated bile acids promoted a more effective lipid absorption. The increased lipid absorption observed in the conventional mice contributed to a higher food efficiency and an increased obesity compared with the germfree mice [29]. In a zebrafish model, it was shown that gut bacteria facilitate the absorption of long-chain and medium-chain fatty acids as well as intracellular lipid droplet formation in enterocytes [59]. Metabolites produced by a Firmicutes strain, which had been isolated from the zebrafish intestine, increased the number of lipid droplets in enterocytes. In contrast, metabolites produced by a Bacteroidetes strain or a Proteobacteria strain did not exhibit this effect [59]. This finding indicates that certain gut bacteria may affect intestinal lipid absorption and lipid droplet formation, whereas others do not. Moreover, the fact that conventional mice display significantly higher small intestinal levels of fatty acid translocase (CD36) than germfree mice suggests that intestinal bacteria mediate an increase in gene and protein expression of this lipid transporter [60]. The relevance of these findings for the development of metabolic disease is not yet clear because research into the role of bacteria in lipid absorption is hampered by the fact that the underlying mechanism is not well understood. In particular the role of proteins such as CD36, fatty acid binding protein (FABP) and fatty acid transport protein 4 (FATP4) in long-chain fatty acid absorption is not entirely clear. The high expression of these proteins in the small intestine and their apical location in enterocytes suggest a role in lipid transport across the brush-border membrane. However, studies using mice deficient in the respective proteins reported conflicting results. The difficulty in gaining a better understanding of their role in lipid absorption and diet-induced obesity development could in part be due to the fact that deletion mutants devoid of any of these genes do not display a specific phenotype because their functions can be compensated by other proteins. Such compensatory adaptations protect genetically modified mice from impaired lipid absorption [61], but impede research on the impact of intestinal bacteria on small intestinal lipid uptake. Currently, administration of fluorescently or radioactively labeled long-chain fatty acids to wild-type gnotobiotic mice seems to be the most promising technique to unravel bacterial effects on intestinal lipid metabolism.

## 8. Impact of Intestinal Microbiota on Regulation of Host Energy Metabolism

When energy intake exceeds energy consumption fat becomes deposited in adipose tissue. Adipogenesis induced by high-fat diets is enhanced when lipoprotein lipase (LPL) is overexpressed in adipose tissue. Under such conditions, the uptake of fatty acids from plasma into adipocytes, their esterification into triglycerides, and finally triglyceride deposition in adipocytes exceeds lipolysis of triglycerides as well as their release from adipocytes and their oxidation in various tissues [62]. The gut microbiota has been proposed to affect fat storage by influencing the level of the circulating angiopoietin-like protein 4 (Angptl4) [26], a secreted glycoprotein [63], which is a downstream target of the nuclear peroxisome proliferator-activated receptor family. ANGPTL4 is also known as fasting-induced adipose factor (FIAF) because fasting causes an upregulation of ANGPTL4 in white adipose tissue and liver [64,65]. ANGPTL4 is a lipoprotein lipase (LPL) inhibitor, which regulates the deposition of triglycerides in adipocytes [66]. By way of inhibiting LPL, ANGPTL4 diminishes lipolysis of triglyceride-rich lipoproteins resulting in increased plasma triglyceride levels and subsequently in a decreased uptake of fatty acids into body tissues [67]. Accordingly, mice over-expressing ANGPTL4 display reduced white fat stores [68]. Conversely, suppression of ANGPTL4 stimulates triglyceride storage in adipose tissue [62]. ANGPTL4 mRNA levels are highest in adipose tissue and liver but this mRNA is also found in other tissues, including small intestine and hypothalamus, but at lower levels [64]. Secretion of ANGPTL4 in the intestine was proposed to substantially contribute to its abundance in plasma [26]. This proposition was based on the observation that intestinal ANGPTL4 mRNA levels in conventional mice were twofold lower than those in germfree mice. This led to the conclusion that the gut microbiota represses intestinal ANGPTL4 secretion resulting in decreased levels of circulating ANGPTL4 and consequently in less inhibition of LPL, *i.e.*, increased LPL activity and fat accumulation. In accordance with this interpretation, the relative increase in body fat in conventional *versus* germfree mice was considerably lower in *Angptl4*^−/−^ mice than in wildtype mice. These results were interpreted to mean that germfree mice are generally protected from diet-induced obesity because they display higher ANGPTL4 levels resulting in LPL inhibition and consequently in reduced fat deposition [26]. Another study also observed higher mRNA levels of ANGPTL4 in intestinal mucosa of germfree *versus* conventional mice, but the plasma protein levels of ANGPTL4 between the two mouse groups did not differ [30]. Similarly, administration of *Lactobacillus paracasei* strain F19 to conventional or germfree mice led to increased plasma ANGPTL4 levels and reduced fat accumulation [69]. This suggests that intestinal bacteria do not necessarily lower circulating ANGPTL4 levels as proposed [26], but that at least some members of the gut microbiota exert opposite effects. These observations and other considerations led to the conclusion that the intestinal mucosa does not contribute substantially to circulating ANGPTL4 levels and, therefore, intestinal ANGPTL4 does not play a role as an LPL inhibitor in adipose tissue of germfree mice [70].

Besides providing additional energy, SCFA also fulfill a regulatory function in host energy metabolism. Acetate, propionate, and butyrate are ligands of the G-protein coupled receptors FFAR2 (free fatty acid receptor 2) and FFAR3 (formerly GPR43 and GPR41); these receptors are expressed in ileal and colonic enteroendocrine L cells, adipocytes and immune cells [71]. Upon activation of FFAR3 by SCFA, adipocytes secrete leptin [72] and enteroendocrine cells secrete peptide YY (PYY) [73]. Both hormones reduce appetite [74]. In primary murine colonic cell cultures acetate and propionate enhance the secretion of glucacon like peptide 1 (GLP-1) by enteroendocrine L cells [75]. GLP-1 stimulates insulin production by pancreatic beta cells, improves insulin sensitivity and promotes satiety. Mice lacking *Ffar2* or *Ffar3* display low GLP-1 levels and an impaired glucose tolerance, suggesting that SCFA play an important role in glucose homeostasis [75].

In a recent human intervention study, propionate delivery to the colon was accomplished by oral intake of 10 g of inulin esterified with propionate [76]. Propionate delivered in this way led to increased levels of plasma PYY and GLP-1 accompanied by a reduced energy intake. When the administration of the inulin-propionate ester (10 g/day) was extended to 24 weeks, weight gain, abdominal adipose tissue and hepatic lipid content were decreased and insulin sensitivity was beneficially influenced compared with the inulin control [76]. However, contrary to the observation made after the ingestion of one dose of the inulin-propionate ester, no differences in PYY or GLP-1 could be detected after administration of this compound over an extended period of 24 weeks compared with the inulin-control. The authors hypothesized that the FFAR2/3 receptor response was desensitized over time and that the observed long-term beneficial effects were not mediated by PYY and GLP-1. Moreover, the role of PYY in obesity *per se* is contradictory: On the one hand, PYY has anorexigenic effects that counteract obesity; on the other hand, it slows down gut transit time. A prolonged retention time of dietary constituents results in an extended fermentation of bacterial substrates and a more complete absorption of nutrients and SCFA, both of which could promote obesity [28] (discussed in [77]).

Intriguing studies by Gilles Mithieux and colleagues identified a novel mechanism that helps to explain the beneficial effects of SCFA in the prevention of diabetes [78,79,80]. Both propionate and butyrate were demonstrated to enhance intestinal gluconeogenesis, which mediates these effects by way of signaling through neural circuits that link the enterohepatic portal system with the brain. In the fasted state approximately 20%–25% of the endogenously produced glucose stems from intestinal gluconeogenesis [80]. The neural system surrounding the portal vein senses the glucose produced by intestinal gluconeogenesis and sends a signal to the brain, which modulates the energy and glucose metabolism. Not only dietary proteins promote intestinal gluconeogenesis but also propionate and butyrate do so by two complementary mechanisms [78]. Butyrate promotes intestinal gluconeogenesis in an FFAR2-independent way: oxidation of this SCFA by enterocytes results in higher ATP levels and, in turn, higher cAMP levels [81]. The latter induce the expression of gluconeogenesis genes. Propionate acts both as a substrate of gluconeogenesis and as an agonist of FFAR3, whose activation enhances gluconeogenesis via a neural gut-brain circuit in the afferent periportal nervous system [78]. Therefore the anti-obesogenic and anti-diabetic effects of fermentable fibers are thought to be mediated by intestinal gluconeogenesis via the fermentation products propionate and butyrate.

## 9. Role of Low-Grade Inflammation in Metabolic Disease

Obesity and type 2 diabetes share a common feature, namely the activation of inflammatory pathways [82]. Gut bacteria have been proposed to be involved in the development of low-grade inflammation in obese and diabetic individuals [83] since they produce pro-inflammatory molecules such as lipopolysaccharides (LPS), flagellins and peptidoglycans. The endotoxin LPS is a cell wall component of Gram-negative bacteria, which becomes liberated into the gut lumen upon bacterial cell lysis [70]. High-energy diets, in particular high-fat diets, as well as the obese and the diabetic phenotype are associated with high plasma LPS concentrations in humans and mice [84,85,86,87]. Chronic infusion of LPS enhances obesity and pro-inflammatory signaling and also reduces hepatic insulin sensitivity in wildtype mice, while mice deficient in the glycoprotein cluster of differentiation 14 (CD14) are devoid of most of the symptoms induced by high-fat diet feeding or LPS infusion [85]. CD14 binds and presents LPS to the receptor complex Toll-like receptor 4 (TLR4)/myeloid differentiation factor 2 (MD-2), which triggers an inflammatory response of the host to the bacteria [85,88]. Hence, LPS and its downstream signaling cascade might be a causal link between the gut microbiota and metabolic disease.

But how does LPS enter the host? It has been proposed that LPS is taken up together with dietary lipids within chylomicrons or via paracellular transport through tight junctions [89]. Therefore, dietary fat might promote LPS uptake from the intestine. In support of this hypothesis, high-fat diet feeding in mice was reported to impair the gut barrier resulting in increased plasma endotoxin levels. Impairment of the gut barrier was possibly due to the down-regulation of certain tight junction proteins. In these mice the high-fat diet-induced changes were accompanied by changes in the gut microbiota [90]. Whether the dietary pattern *per se* increases intestinal permeability or whether it alters microbiota composition, which in turn impairs the gut barrier function, remains to be clarified. Presumably, an increase in gut permeability in conjunction with an LPS-enriched gut microbiota, both triggered by high-fat diets, facilitate LPS absorption and contribute to the development of low-grade inflammation.

LPS absorption could also be increased by activation of the endocannabinoid system by LPS itself. In mouse macrophages, LPS promotes the formation of anandamide, a physiological ligand of the endocannabinoid receptor 1 [91]. In mice, activation of this receptor increases gut permeability and plasma LPS levels [92]. In conclusion, a high-fat diet may change the intestinal microbiota in favor of LPS containing bacteria, which in turn may cause activation of the endocannabinoid system, associated with a weakening of the gut barrier and resulting in increased LPS absorption. This vicious cycle could promote the development of low-grade inflammation under conditions of high-fat feeding.

However, a recent study using three mouse strains differing in their genetic background and their propensity to develop obesity did not reveal any effect on gut barrier integrity in response to four weeks of high-fat feeding (48 kJ% plant fat) in any of the mouse strains. Even prolonged high-fat diet feeding for 12 weeks with a higher fat content (60 kJ%) did not impair small intestinal and colonic permeability in BL/6J mice. These mice displayed an intact intestinal barrier function and inconspicuous LPS levels in portal vein plasma even when fed a high-fat diet based on 78 kJ% lard [93]. Furthermore, owing to the fact that Firmicutes, which do not produce LPS, are enriched in obese subjects, LPS as the microbial mediator of endotoxemia in metabolic disorders is counterintuitive. However, Firmicutes produce fructanases, which degrade fructans to fructose. Following its absorption into epithelial cells fructose becomes phosphorylated by ketohexokinase. Subsequent depletion of intracellular ATP and phosphate levels transiently interrupts protein synthesis and/or increases oxidative stress, which in turn may reduce tight junction protein expression and thereby increases gut permeability and endotoxemia [94]. Whether Firmicutes do indeed contribute to an impairment of the gut barrier by this or another mechanism and thereby facilitate the uptake of LPS derived from the diet or from Gram-negative gut bacteria has not yet been investigated.

## 10. Obesogenic and Anti-Obesogenic Intestinal Bacteria

Apart from phylum level changes, microbiota modifications at the class, family and genus level are reported for obese subjects. For instance, the bloom of Erysipelotrichi in an obese human individual [95] and in obese mice [30,32,37] suggests a contribution of members of this bacterial class to obesity. The presence of a member of the Erysipelotrichi, *C. ramosum*, was linked to symptoms of the metabolic syndrome in women with type 2 diabetes [96]. Another human study confirmed an association between obesity and an increased intestinal abundance of this species [97], suggesting that *C. ramosum* is critically involved in obesity development. Recently the presence of this bacterium in gnotobiotic mice harboring a simplified gut microbiota of human representative species promoted body weight gain and body fat deposition during high-fat diet intervention [55]. The absence of *C. ramosum* from the microbial community reduced the severity of high-fat diet-induced obesity. The mechanism underlying this obesogenic effect possibly involves the up-regulation of genes playing a role in glucose and lipid absorption as well as in intracellular lipid storage in the small intestine (*Glut2*, *Cd36*, *Plin2*) (Figure 2) [55]. However, whether the up-regulation of these genes does indeed result in increased nutrient absorption and whether this causally contributes to obesity as proposed by Woting *et al.* [55] requires further mechanistic research involving the use of labeled glucose and lipids.

Fei and Zhao (2013) described another obesogenic, LPS-containing bacterium, *Enterobacter cloacae* B29 (*E. cloacae*), which they isolated from an obese Chinese patient. When introduced into germfree mice, these mice developed low-grade inflammation and obesity under high-fat diet feeding; these symptoms were less severe in germfree mice fed the same diet [98]. It appears that more than one strain in the complex and diverse human gut microbiota is capable of promoting obesity in mice. Indeed, eight of twelve human gut bacterial strains, including *Bacteroides* strains and one member of the Proteobacteria (*Escherichia coli*), increased adiposity when introduced as monocultures into germfree mice fed a low-fat, polysaccharide-rich diet. The most pronounced effect on adiposity development was observed after associating germfree mice with either *Parabacteroides distasonis* or *Bacteroides vulgatus*, both isolated from a human volunteer [99]. However, the relevance of single bacterial strains on obesity development needs to be verified in more complex microbial communities or even in conventional mice because the obesogenic properties of a single bacterium may get lost when a conventional background microbiota is present, as observed for *E. cloacae* B29 [98].

An anti-obesogenic effect was reported for the mucin-degrading bacterium *Akkermansia muciniphila* (*A. muciniphila*) [100]. High-fat diet feeding *per se* reduced the cell count of *A. muciniphila*, whereas the treatment with oligofructose or grape polyphenols stimulated the growth of *A. muciniphila* in mice and reduced adiposity and metabolic endotoxemia [100,101]. Also in humans was a high abundance of this species associated with a healthier metabolic status and improvements in insulin sensitivity and blood cholesterol levels after calorie restriction [102]. In support of these studies, treatment of high-fat diet-fed mice with viable *A. muciniphila* improved gut barrier function and reversed diet-induced obesity, insulin resistance and endotoxemia [100]. Concordantly, the oral gavage of *A. muciniphila* to high-fat diet-fed mice improved glucose tolerance, attenuated visceral white adipose tissue inflammation by increasing the number of regulatory T cells and reducing the levels of pro-inflammatory cytokines, and restored the number and density of mucus-producing goblet cells similar to effects that occurred after the administration of the anti-diabetic drug metformin [103]. These studies identified the potential of *A. muciniphila* to restore glucose tolerance under high-fat diet conditions in conventional mice with a complex microbiota.

Probiotic bacteria, in particular species belonging to the genera *Lactobacillus* and *Bifidobacterium*, or *Escherichia coli* Nissle 1917 are being used to prevent or treat gastrointestinal disorders [104]. Therefore, the administration of probiotics might also offer the chance to prevent or even treat obesity and diabetes. However, the reported effects of certain *Lactobacillus* spp. on body weight vary considerably. A meta-analysis indicated that *Lactobacillus acidophilus*, *Lactobacillus fermentum*, and *Lactobacillus ingluviei* promote weight gain, while the administration of *Lactobacillus plantarum* (*L. plantarum*) and *Lactobacillus gasseri* (*L. gasseri*) is associated with weight loss in obese humans and animals [105]. Oral application of a diet supplemented with two *Lactobacillus* strains (*Lactobacillus curvatus*, *L. plantarum*) to obese mice reduced obesity and improved inflammatory markers in adipose tissue [106] while the administration of *L. plantarum* alone attenuated body weight gain and dyslipidemia in high-fat diet-fed mice [107]. The consumption of fermented milk containing *L. gasseri* significantly reduced BMI, body fat mass, and waist and hip circumference in healthy subjects with large visceral fat depots [108]. These effects were attenuated after the consumption of the milk product was stopped, suggesting that probiotics must be consumed continuously to maintain their anti-obesogenic effects.

The inverse relationship between the size of the bifidobacterial population and the incidence of metabolic disease suggests that *Bifidobacterium* spp. have an anti-obesogenic or anti-diabetic potential [109,110,111]. Indeed, supplementation of a high-fat diet with oligofructose restores the number of intestinal bifidobacteria and reduces symptoms of metabolic diseases [112,113]. Therefore, bifidobacteria were hypothesized to mediate the oligofructose-induced improvement of various symptoms of the metabolic syndrome. However, in mice associated with a defined microbial community and fed a high-fat diet, the beneficial effects of oligofructose were independent of the presence or absence of *Bifidobacterium longum* [114]. Oligofructose *per se* reduced obesity and improved glucose tolerance. In contrast, mice mono-associated with *Bifidobacterium animalis* (*B. animalis*) and germfree control mice gained less body weight on a high-fat diet compared with mice mono-associated with *E. cloacae*, indicating that *B. animalis* was less obesogenic than *E. cloacae*. Actually, both *B. animals*-associated mice and germfree mice were not protected from diet-induced obesity as they displayed similar body weights (approximately 33 g and 36 g) after 10 weeks of high-fat diet feeding. In conclusion, *B. animalis* does neither promote nor prevent obesity development [98]. Unlike the *B. animalis* strain used by Fei and Zhao, the daily gavage of *B. animalis* ssp. *lactis* 420 to high-fat diet-fed mice as well as the administration to mice of a high-fat diet pre-mixed with *Bifidobacterium breve* B-3 (*B. breve* B-3) alleviated diet-induced obesity [115,116]. Also in humans, the daily intake of a capsule containing the lyophilized powder of *B. breve* B-3 reduced body fat mass [117]. It may be surmised that the anti-obesogenic effect of *Bifidobacterium* spp. is species- or even strain-specific. It also needs to be clarified whether the animal data are of relevance for the human situation.

Taken together, animal studies suggest that species such as *C. ramosum* and *E. cloacae* are associated with symptoms of metabolic disease, whereas species such as *A. muciniphila* and strains of *Lactobacillus* spp. and *Bifidobacterium* spp. are linked to beneficial effects. However, it is still unclear how these bacteria trigger the observed effects and by which molecules they are mediated.

## 11. Conclusions

The digestive tract represents a complex microbial ecosystem. Associations between certain diseases and patterns of microbiota composition are in part inconsistent among studies, and their meaning is mostly unclear. Moreover, methodological and population-based differences between studies are much larger than the biological differences between obese and lean subjects within a given study, highlighting the need for harmonization and standardization of study designs, sample preparation and analysis. However, even though the reported microbial changes in response to interventions are not uniform, the reduced microbial diversity in metabolically diseased patients seems to be a fairly recurrent finding. Which bacterial molecules are absent or present in a less diverse microbiota and thereby mediate the development of metabolic diseases is unknown. Gnotobiotic animal models offer the opportunity to investigate the interaction of potentially obesogenic and anti-obesogenic bacteria and their metabolites with other gut bacteria and the host. Their known microbial status circumvents the drawbacks of a complex and inter-individually different microbiota in conventional animals and humans. Once the molecular mechanisms underlying the obesogenic and anti-obesogenic effects of gut bacteria have been elucidated, investigations in more complex animal models and finally in human subjects will help to clarify the relevance of these findings.
